# Involving children in global health policy and programming: practical guidance to get started

**DOI:** 10.1093/heapro/daaf041

**Published:** 2025-04-23

**Authors:** Gabrielle Mathews, Bethany Jennings, Sarah Sterlini, Audrey Prost, Almaaz Mudaly, Kenneth Kwok, Jason Cohen, Irene Rizzini, Maree Foley, Sonora English, Srivatsan Rajagopalan, Toyyib O Abdulkareem, Sarah L Dalglish

**Affiliations:** Children in All Policies 2030, Institute for Global Health, University College London, 90 Tottenham Court Road, London W1T 4TJ, United Kingdom; Children in All Policies 2030, Institute for Global Health, University College London, 90 Tottenham Court Road, London W1T 4TJ, United Kingdom; Children in All Policies 2030, Institute for Global Health, University College London, 90 Tottenham Court Road, London W1T 4TJ, United Kingdom; Children in All Policies 2030, Institute for Global Health, University College London, 90 Tottenham Court Road, London W1T 4TJ, United Kingdom; Children in All Policies 2030, Institute for Global Health, University College London, 90 Tottenham Court Road, London W1T 4TJ, United Kingdom; KIDsforSDGs, Hong Kong, China; KIDsforSDGs, Hong Kong, China; Pontifical Catholic University of Rio de Janeiro, Rua Marquês de São Vicente, 225, Gávea - Rio de Janeiro, RJ 22451-900, Brazil; Children in All Policies 2030, Institute for Global Health, University College London, 90 Tottenham Court Road, London W1T 4TJ, United Kingdom; Institute for Global Health, University College London, 90 Tottenham Court Road, London W1T 4TJ, United Kingdom; Children in All Policies 2030, Institute for Global Health, University College London, 90 Tottenham Court Road, London W1T 4TJ, United Kingdom; NCD Alliance, 31-33 Avenue Giuseppe Motta, 1202 Geneva, Switzerland; Children in All Policies 2030, Institute for Global Health, University College London, 90 Tottenham Court Road, London W1T 4TJ, United Kingdom

**Keywords:** youth engagement, youth involvement, global health, participation, rights-based approach, child health

## Abstract

Children face new and growing threats to their health and well-being, including rising rates of non-communicable diseases and mental health disorders linked to the influence of commercial determinants of health and climate change, among other issues. Yet despite their right to participation established under the United Nations Convention on the Rights of the Child, children are rarely invited to participate in global health processes meant to benefit them, whether by selecting priorities, designing policies and programming, advocating for their adoption or evaluating their implementation or impact. We call for greater involvement of children in global health initiatives, particularly those designed to benefit them, and lay out five principles to structure such engagement: (i) respect for children’s right to participation, (ii) protection of children’s safety and well-being as a foremost concern, (iii) age-appropriate interactions, (iv) reasonable inclusivity, and (v) transparency and accountability towards child participants. We provide practical recommendations for engaging with older children based on our experience with the Youth Advisory Board of the Children in All Policies 2030 initiative, which included 22 children aged 13–18 from 17 countries who provided input across all areas of our work. Finally, we show the benefits of engaging with children for organizations, the impact they seek to achieve, and child participants themselves.

**Contribution to Health Promotion**

- Considers how children and adolescents under the age of 18 are rarely invited to engage in health policymaking and program design, despite this being their right under widely ratified human rights treaties.
- Imparts lessons and guidance on how to safely and effectively foster meaningful participation of older children.
- Shares practical examples of implementing age-appropriate and accessible interactions, fostering inclusivity, and ensuring transparency and accountability.
- Demonstrates how engaging children and adolescents not only fulfils their rights but also substantively improves policy and programming.

## INTRODUCTION

Children, defined as people under 18 years of age, face an uncertain future with many threats to their health (Clark et al. 2020). Child rights-based approaches to policy and programming, including engaging with children directly, provide a meaningful route to responding to these threats (Save the Children 2012). The United Nations Convention on the Rights of the Child (UNCRC) is the world’s most widely ratified human rights treaty. Under the UNCRC, children have the right to express their views in decisions that affect them; however, their participation is often not realized in practice. With few exceptions, children are not consulted in policymaking, political processes, global governance or other spheres of decision-making (Tisdall and Cuevas-Parra 2020). In the field of global health, there have been increasing calls to involve children in deliberation, planning, and decision-making processes (Bulc et al. 2019, Lal et al. 2019, WHO 2022). However, substantial barriers have prevented these calls from being fully realized, including tokenism, adults’ lack of accountability, and cultural misconceptions that children do not have anything valuable to contribute (Lundy 2018, Collins et al. 2021). These problems are exacerbated by the limited timeframes in which policymaking and programme design occur, further constraining meaningful engagement (Percy-Smith and Thomas 2009).

Models of child participation differ in how they define childhood and conceptualize children’s involvement. Roger Hart’s influential ‘ladder of participation’ problematizes the balance of power between adult and child stakeholders (Hart 1992). An ecological life course approach emphasizes the interconnectedness of children’s health and well-being from childhood into adulthood and contextualizes it within enabling environments for health, shifting the focus from simply hearing children’s views to engaging children and their communities in developing such environments (Were et al. 2015). In this view, children are not assumed to have the same capacities as adults but supportive relationships help develop their competencies in an atmosphere of respect (Atwool 2006). Children’s participation thus brings growth not only to children but also positively impacts adult participants and the environments they co-create together.

Engaging children in decision-making processes leads to more effective and sustainable outcomes by shaping policies and programs that better address their needs (Checkoway 2011, UNICEF 2020, Rizzini 2024). Numerous examples across different levels of policy and decision-making demonstrate the success of open dialogue, where children can share their knowledge and experiences with adults who are committed to acknowledging and using their inputs (Cuevas-Parra and Tisdall 2019). In Karnataka state in southwest India, children were supported to help develop 5-year plans for 56 *gram panchayats* (village councils), resulting in the inclusion for the first time of environmental concerns and issues facing people with disabilities, in many cases rejuvenating moribund *panchayat* processes (Concerned for Working Children 2004). In Australia, as part of the city planning process, Naarm (Melbourne) used innovative methods to engage children aged 3–12 years, such as a ‘walkabout’ that resulted in modifying the timing of pedestrian road crossing lights to enable children and others to cross safely (Kotsanas et al. 2014). In Ghana, school-aged children helped update the country’s early childhood care and development policy by methods including an art contest and a video; their suggestions on improved school sanitation and expanded provision of community centres and libraries were included in the final policy (Akofio-Sowah et al. 2024). For adults willing to listen, there is no child too young to engage: a study on the participation rights of premature babies shows that children of all ages can express their views regarding their experiences (Alderson et al. 2006).

In this Perspective piece, we argue for including children under the age of 18 in the conception, design, and evaluation of global health policy and programming. We draw on our experience in the Children in All Policies 2030 initiative, created in 2021 to implement the recommendations of the WHO-UNICEF-*Lancet* Commission (Dalglish et al. 2021). With reference to key principles and the literature on effective child participation, we offer practical recommendations for those seeking to start engaging with older children and adolescents and outline the challenges and benefits of including children’s voices and perspectives in global health initiatives.

### The Youth Advisory Board of the Children in All Policies 2030 initiative

In 2021, the Children in All Policies (CAP-2030) initiative was founded to implement the recommendations of the WHO-UNICEF-*Lancet* Commission report ‘A future for the world’s children?’ (Clark et al. 2020). Amongst these recommendations was that children should be given ‘high-level platforms’ to share their concerns and ideas, as part of a broader movement towards children’s participation in policy and decision-making. As such, in 2023, 22 young people were selected from over 90 applicants, with a view to ensuring balanced membership in terms of geography, gender, and background. The name of the Youth Advisory Board (YAB) was selected to emphasize participants’ maturity rather than their status as legal minors. YAB members were aged 13–18 years and hailed from 17 countries; all were conversant in English, the YAB’s working language.

Over the course of a year, the YAB met virtually in small groups and larger convenings to provide input on CAP-2030’s core areas of work, including climate change, harmful commercial marketing, data for decision-making, and racism and child health. They provided input in a variety of formats, including by writing blogs, creating PowerPoint presentations, participating in surveys and focus group discussions, and speaking to decision-makers at the World Health Organization, UNICEF, and other major international bodies. YAB members have served as named authors on scientific publications and spoken in large public forums, sharing their experiences and perspectives. Their contributions have substantively shaped the outputs, communications and direction of CAP-2030’s activities.

## GUIDING PRINCIPLES AND PRACTICES FOR ENGAGING WITH CHILDREN

### Child participation is a human right

The right (not the obligation) of children to express their views is enshrined in Article 12 of the UNCRC, and further elaborated in the treaty’s General Comments, as the ‘ongoing processes which include information sharing and dialogue between children and adults based on mutual respect, and in which children can learn how their views and those of adults are taken into account and shape the outcome of such processes’ (Committee on the Rights of the Child 2016). The legal and practical implications of these clauses remain a matter of considerable debate, which are also shaped by national legal frameworks.

Given that children’s right to express their views is hardly realized in most global health policy, programming, and planning decisions, we considered that any move in this direction would be an improvement. We entered the practice arena by creating the YAB as a platform to engage older children in ongoing processes related to our initiative. Engaging with children in global health using a rights-based approach ideally redistributes power amongst stakeholders. As such, we invited YAB members to appraise and comment on our priorities as an initiative and interact with our Executive Committee to evaluate our work and progress against objectives. Children had already shaped our strategic direction via their input into the WHO-UNICEF-*Lancet* Commission and its 2020 report through a series of focus group discussions on four continents (Clark et al. 2020).

### The safety and well-being of the child is paramount

Global health organizations seeking to engage with children must integrate child safeguarding into all policies, procedures, and practices, which establishes a foundation of trust and security and protects both the organization and the children it engages with. The ‘International Child Safeguarding Standards’, developed by the Keeping Children Safe Coalition, designed through a global network operating in 120 countries, provide useful guidance (Keeping Children Safe Coalition 2024). Organizations must invest time and effort into considering how they will work with children, the specific risks involved, and how these will be mitigated. Developing policies and ensuring staff engaging with children are appropriately trained is both labour and resource intensive: organizations will benefit from hiring a dedicated individual with full background checks who can oversee the process; however, the responsibility for keeping children safe is shared by all.

Two part-time staff members of our UK-based secretariat were responsible for the YAB; they began by reviewing organizational guidance and writing a safeguarding policy that covered all aspects of our work in line with UK standards of safeguarding and data protection. Since we work across borders, we came to a written agreement with partners about which standards would prevail when national regulations differed. We also developed an online behavioural code of conduct (for children and adults) and a privacy notice, both written in child-friendly language, outlining how conflicts and inappropriate behaviours would be managed and noting that participants had the right to withdraw at any time (Supplementary materials). When working with children in online environments, it may be unclear who is best placed to report and handle disclosures or other issues—so this must be specified in advance, including highlighting lines of reporting should any concerns arise.

Children joining the YAB required signed permission slips from parents, who were also involved in the introductory interview, with additional parental consent for each activity. All interactions with children (which were virtual) were conducted in the presence of two or more adults. We only used online communication platforms with appropriate safety and privacy features, although this excluded some well-known apps. Importantly, we also sought to make participation in the YAB a positive experience by making interactions fun and being respectful of children’s time. We asked what they would like to learn about, connected them with experts, and tailored experiences according to their preferences.

### Interactions must be age-appropriate and accessible

To ensure that the voices of as many children as possible are heard, it is important that approaches to child participation are age appropriate. Many programmes for engaging children or youth begin around age 13, often extending to involve 18–25-year-olds. Given the wide variability of capabilities across and within this age band, we recommend creating as narrow age bands as is practically feasible. We proposed a panoply of activities and ways for children to participate, including virtual meetings, surveys, focus group discussions, online bulletin boards, opportunities to present, and creative pursuits, including video and audio projects, which could be engaged with both synchronously and asynchronously. Methods of involvement should be co-developed with children so they can express themselves via their preferred mediums (Lansdown 2011).

Working with young people remotely can present difficulties for age-appropriate interactions. Adult facilitators must keep in mind that school-age children have considerable school and family obligations, which must take precedence. As such, activities should not be timed during school hours or overnight, which can create planning difficulties for advisory boards bringing together members across continents. We offered morning and evening sessions for each meeting, with no pressure to join and no consequences for missing meetings. Meeting recordings and outcomes were always shared afterwards. For presentation tasks, particularly in public forums or with higher-level stakeholders, we provided dedicated preparation sessions and additional language support when required; we also planned for problems with technology and internet access. Language and communication methods must be selected so that they are accessible to everyone, such as by avoiding overly technical language and ensuring materials are accessible to disabled people and ideally in multiple languages (WHO 2022).

### Inclusivity is critical but should not be a barrier to beginning engagement

Modes of child participation that are relatively easier for global health organizations to manage may also be among the least inclusive. We were aware that most children who participated remotely in an advisory board would be of relative global privilege given that they are likely to speak English and have access to a computer and an internet connection. The concerns such children raise may be far from those of the much larger group of children from poorer, marginalized, and historically oppressed populations. When constituting our YAB, we were unable to overcome this problem but were careful to select participants from a range of countries and ensure the group was not dominated by a global minority.

Global health stakeholders concerned about reifying historical inequities should see YABs as just one of many ways to engage with children globally, alongside other forms in which children from less privileged milieux are able to participate. Leading child participation expert Laura Lundy warns against making the perfect the enemy of the good: ‘Participation should not be rarefied to the point that it is considered unattainable…tokenism is sometimes a start’ (Lundy 2018). In addition to our YAB, we have also engaged with children through country-specific work, such as in Nepal, where they participated in a citizen project on nutrition and climate change, and in Ghana, where they helped revise the national early childhood care and development policy (Hoernke et al. 2023, Akofio-Sowah et al. 2024).

### Adults owe children transparency and accountability

When engaging with children, honesty regarding their role is critical. To what extent will their inputs actually influence the strategic or operational direction of the organization? In what concrete ways did their input make a difference? Beyond merely listening to children’s views, it is crucial to demonstrate that actions have been taken accordingly, an ethical imperative that takes on added dimensions when working across Global North and Global South contexts (Powell et al. 2023). Transparency and trust between the young people and the facilitating organization are best achieved through maintaining clarity regarding the role and expectations of young people and the topics that are within the scope of their influence. We kept YAB members apprised of how their inputs were used and how they shaped the different processes they were involved in; we also changed activities in response to their feedback and introduced new ones, such as making short videos.

Formal feedback mechanisms should be embedded into child participation processes so it is ‘uncomfortable for adults to solicit children’s views and then ignore them’ (Lundy 2007). This productive discomfort can be created by outlining the scope at the outset, with the additional benefit of managing children’s expectations so that they can calibrate their engagement accordingly. Clear expectations can also relieve the pressure on adult professionals, who carry the burden of balancing the voices and inputs of children against the desires of other key stakeholders such as board members, funders, and the like. We tried to be as clear as possible about children’s scope for shaping the direction of our work at the outset; we also conducted an end-of-year ‘celebration’ where we fed back exactly how the YAB had affected and improved our work and recognized their achievements.

## BENEFITS OF AN INVESTMENT IN CHILDREN’S PARTICIPATION

While evidence is still accumulating, investment in children’s participation in global health initiatives and policymaking has major potential benefits: it improves the organization’s programming, maximizes impact, and provides substantial positive outcomes and opportunities for child participants (Bulc et al. 2019, Joining Forces 2021). Our experience suggests that children’s participation has major underused potential to improve the quality and impact of a global health organization’s work—especially when participation is meaningful and carries through from the specific project’s conception to its completion and dissemination. CAP-2030’s YAB was invited to participate across all areas of the initiative’s work, leading to meaningful modifications in emphasis, direction, and impact (Table 1).

**Table 1.** Involvement of Children in All Policies 2030’s YAB across areas of work.

Project	Role of YAB	How the work was shaped by the YAB’s participation
Policy report on harmful commercial marketing to children and youth, with NCD Alliance	Participated in focus group discussions about their concerns about marketing, flaws in current regulations, and potential new policies Reviewed and commented on draft versions of the report Helped launch the report at the Global Forum for Adolescents	Broadened the scope of harmful industries being considered Increased emphasis on mental health outcomes Provided real-life experience about being marketed to, impacts on consumption patterns, etc. Increased impact and reach by boosting dissemination to youth-focussed organizations
Research project on racism and child health globally	Responded to a survey on priorities for the research project at its outset Reviewed and provided feedback on the interview guide for focus groups discussions with children globally	Substantially shaped the interview guide, resulting in a more nuanced and intersectional view Produced public-facing work (e.g. blogs) exploring under-explored facets of this topic
WHO-UNICEF Child Health and Well-being Dashboard	Reviewed the dashboard indicators Presented to a global expert group at a meeting hosted by the World Health Organization	Influenced WHO priorities for future monitoring efforts Suggested indicators for a future ‘shadow’ dashboard
Scientific publication on priorities for future research on climate change and child health	Participated in discussions about emerging research priorities Reviewed a manuscript for publication in a peer-reviewed journal	Shaped discussion of the outcomes of an evidence gap map Provided direction for areas of future research and action, including by identifying new areas of work
Learning Planet Festival, Global Learning Week	Presented on solutions to tackle global challenges such as intergenerational poverty and digital inclusion Received support from CAP-2030 and KIDSforSDGs to focus their ideas and present them clearly	Improved connections to a plethora of leading global youth-focussed organizations Created greater visibility on topics of importance to today’s youth

As one illustrative example, YAB members helped develop a policy report on the marketing of harmful products to children and young people globally (CAP-2030 and NCD Alliance 2023). Input from YAB members increased emphasis on mental health outcomes and provided depth to the report via their lived experiences, as well as concrete suggestions for policy interventions that went beyond experts’ recommendations (Soraghan et al. 2023). YAB members also increased the reach and impact of the report by boosting dissemination through their own youth-focussed networks (the YAB page is the second-largest driver of new traffic to the CAP-2030 website). As part of the consultation process, children were brought in early, before the outline of the report was established. Nonetheless, we discovered that involving the YAB even earlier, during the initial scoping of the review, would have been preferable, because their suggestions of additional harmful industries to focus on (namely the beauty, skin whitening, and nutritional supplement industries) were not able to be fully incorporated.

Other global health initiatives have found similar benefits to engaging children and young people early in their processes. For example, consultations under the UN Major Group for Children and Youth and the Partnership for Maternal, Newborn, and Child Health (PMNCH) have shaped emerging frameworks of adolescent well-being to render them more multi-dimensional, transcending physical health to encompass evolving capacities and lived experiences (Mohan et al. 2022). UNICEF has developed multiple tools and pathways for children’s participation, including the U-Report application, which it has used to gather input towards achieving its strategic priorities across many areas of its mission. Other examples of children’s participation and its benefits are available at national and local levels; while these can provide inspiration for global health stakeholders, frequently such interventions use more consultative and less empowering modes of engagement, which should be strengthened to capture all the potential benefits (Larsson et al. 2018).

When done sensitively, involving children in global health policy and programming also accrues benefits to child participants themselves. Members of our YAB had access to development opportunities in data analysis, writing and presenting, and networking opportunities with each other and experts in the field. In some cases, children were connected to opportunities for formal training. Other benefits included certificates of participation and recommendation letters for further study. YAB members provided positive feedback about their experience, using words like ‘community’, ‘encouraging’, ‘inspiring’, and ‘learning experience’ in an interactive feedback session, and many have continued to collaborate with CAP-2030 beyond the length of their initial engagement.

The advantages of training opportunities beyond the school walls are well documented, as are the benefits of children having a trusted adult beyond the family (Whitehead et al. 2019, Frederick et al. 2023). Participation has been shown to provide children with developmental benefits, such as developing competencies and confidence to play an active role in society (DFID–CSO Youth Working Group 2009). Participants have also been shown to develop transferrable communication skills and improved awareness of their rights, including their right to participate in decisions that affect them (Lansdown 2001, UNICEF 2020). To ensure benefits to children are realized and their participation is ethical and effective, evaluation frameworks should measure the scope of participation (at which point they are included, the level of engagement, and degree of inclusivity), the quality of participation (whether it is meaningful and ethical), and the outcomes (the impact on the children and adults involved and on the relevant programme or policy) (Lansdown and O’Kane 2014).

## CONCLUSION

Involving children in global health policy and programming is a moral imperative and a strategic necessity for creating sustainable and effective initiatives. This Perspective underscores the importance of amplifying the voices of children, who are often excluded from conversations that impact their futures. Inclusion can take many forms, beginning modestly with a view to continuously strengthen engagement, rather than being impeded by concerns of tokenism. We provide practical guidance on how to get started with involving children in organizational policy and programming. Through CAP-2030’s YAB, we have seen firsthand the transformative potential of child participation: these young individuals provided unique insights and perspectives that challenged traditional models of policymaking, leading to more inclusive and effective solutions. However, meaningful engagement requires a commitment to safeguarding, accountability, and transparency, and a significant resource investment. By embedding children’s views and perspectives, we not only empower young people but also promote improved global health policies and programmes that are representative of those they aim to serve. The future of global health depends on the active participation of today’s children.

## Supplementary Material

daaf041_suppl_Supplementary_Material

## Data Availability

Most of the data underlying this article is available in the article and in its Supplementary Material. Some data about the membership of the Youth Advisory Board cannot be shared publicly to protect the privacy of participants under the age of 18; this data will be shared on reasonable request to the corresponding author.
