# Supplementary material for: Involving children in global health policy and programming: practical guidance to get started
Source: Health Promot Int. 2025 Apr 23;40(2):daaf041. doi: 10.1093/heapro/daaf041 (PMC12015606; doi:10.1093/heapro/daaf041)
Supplement: daaf041_suppl_Supplementary_Material [file daaf041_suppl_supplementary_material.zip › Online_Behaviour__Code_of_Conduct_CAP-2030_vf_SHARE.pdf]

Code of Behaviour and Safety information for Online Activities

Who is this for?

- Code of behaviour for young people participating in online activities with CAP-2030
- Key information for parents and carers

Key Contacts

CAP-2030 Activity Supervisors
[contact information]

UCL Safeguarding Leads
[contact information]

Behaviour Policy

- Act responsibly. Follow instructions from UCL staff
- Follow housekeeping instructions from the session leader/meeting chair/supervisor that will be given at the start of the session or beforehand. For example, if asked to mute your microphone or turn off your camera, please do so. Repeated failure to follow the instructions will result in you being removed from the session/meeting
- Do not share the invitation link to the online session with others.
- Do not meet with a member of UCL member of staff on your own. Make sure you copy your supervisors to all email correspondence and meeting invitations.
- Engage with the session. Listen and contribute your thoughts and questions at the appropriate times.
- Give yourself time to log in and join the session/meeting on time. Tell us if you can no longer attend.
- Do not attend the UCL session under the influence of alcohol, cigarettes or illegal drugs.

- Only use MS Teams and/or your UCL email address (if possible) for communications with UCL staff members.
- Don’t try to contact UCL staff and students or other participants through their personal emails or social media accounts.
- UCL staff and students or your fellow participants must not contact you through their personal email or social media accounts.
- If a member of staff, student or a participant contacts you in this way, please tell UCL or CAP-2030 staff member about it or tell your parent/carer so they can contact UCL/CAP-2030
- You must not take photographs of the screen or share any images of the online session.

Anti-bullying policy

Online bullying can take different forms, such as:

- Verbal – name calling, making nasty jokes, comments or threats
- Emotional – excluding or ignoring people
- Cyberbullying - sending bullying messages, pictures or videos through social media, texts, email etc.

UCL promotes equality and diversity. We want all young people attending programmes to feel welcome and safe at UCL.

- We will not tolerate any form of bullying.
- You must show respect and kindness UCL staff, students and other young people working with CAP-2030
- You must not make remarks that could upset someone based on their gender, sexuality, ethnicity, disability, religious beliefs or lack of religious beliefs.
- If you notice that someone else is being bullied inform your supervisor or another UCL staff member as soon as possible.
- If you feel upset by the way someone on the programme has treated you, get in touch with a UCL staff member, or your parent or carer and ask for help.

Serious misbehaviour

- In the event of serious misbehaviour, such as bullying, attending a session under the influence of drugs or alcohol or using offensive or inappropriate language or images you will not be allowed to continue with the session or the rest of the programme (if applicable).
- We will contact your parent / carer to discuss it.

What to do if you are worried about something

If you feel worried about anything while you are attending a UCL session or meeting please contact your supervisor (listed at the start of this document) or another member of UCL staff

If you feel uncomfortable about something at UCL, but don't feel able to tell a UCL staff member about it, please talk to a trusted adult (this might be your parent, carer, someone in your family or a teacher at school) and ask for their help.

Information for parents and carers

Health, safety and supervision

We take the safety and privacy of young people very seriously. Please read UCL's Safeguarding children and adults at risk Policy and Procedure for further information and reporting procedure

Online interaction with young people is always supervised and communications with UCL staff will only take place via MS Teams or using the UCL email account if provided. The supervisor(s) is to be copied into all correspondence.

All our activities have risk assessments in place.

Photography, video and social media

UCL students and staff are all instructed not to contact or connect with young people through personal email or social media accounts.

Participants must not take photographs or videos of the screen or share any images of the online session.

Contacting us if you have a concern

If you have any concerns or questions, please contact the UCL staff member acting as supervisor:

CAP-2030 Activity Supervisors
[contact information]

If you need to report a child protection concern about any child related to their attendance at a UCL activity, please contact the following people:

UCL Safeguarding Leads
[contact information]

For further guidance please click on the link: https://www.ucl.ac.uk/human-resources/safeguarding-children-and-adults-risk-policy-and-procedure-staff-and-students

It is important that we stay safe when engaging in online activities and to be aware of the potential risks. We recommend the safety guidance below for further information:

For children

- NSPCC Staying Safe Online
- NSPCC Guidance on how to feel good on social media

For Parents

- NSPCC Online Safety Guides for parents
- NSPCC Guidance on keeping children safe on social media

If at any point, you feel unsafe engaging in online activities associate with the CAP-2030 YAB, please contact a member of our staff and/or tell a parent/guardian or a trusted adult. **Your safety, health and well-being is our priority.**

This document was developed in part based on the Online Code of Behaviour and Safety Information for Online Activities created by the UCL Access and Widening Participation Office.
