# Supplementary material for: Involving children in global health policy and programming: practical guidance to get started
Source: Health Promot Int. 2025 Apr 23;40(2):daaf041. doi: 10.1093/heapro/daaf041 (PMC12015606; doi:10.1093/heapro/daaf041)
Supplement: daaf041_suppl_Supplementary_Material [file daaf041_suppl_supplementary_material.zip › CAP-2030_Safeguarding guidance_FORMATTED.pdf]

Child Safeguarding Guidance

1. Introduction to the safeguarding guidance

1.1 Introduction to CAP-2030 and purpose of safeguarding guidance

CAP-2030 exists to centre children's wellbeing and health at the heart of all policies. As we strive to create a better world for children globally, it is essential that we address the safety and wellbeing of the children we have relationships with, either through direct interaction or partnerships. CAP-2030 is committed to being a safe organisation for all children.

The purpose of this guidance is to outline a framework and agreed code of conduct to ensure that we are a safe organisation, do not cause any child harm, and respond appropriately to any concerns regarding children's wellbeing that may arise. Furthermore, it sets out our standards and expected practices for CAP-2030's partners and networks.

1.2 Whom the safeguarding guidance applies to

Children are defined as anyone under the age of 18 years old in the UN Convention on the Rights of the Child (UNCRC). This guidance applies to any child who interacts with CAP-2030.

All employees, volunteers, interns, consultants, and partners are expected to uphold and carryout the principles and procedures described in this document.

1.3 UCL Policy

This document provides specific guidance for CAP-2030's activities and is to be followed in concurrence with UCL's existing policy. The UCL Safeguarding Children and Adults at Risk Policy and Procedure, should be understood and followed.

Summary of the UCL Safeguarding Children and Adults at Risk Policy and Procedure

The Policy focuses on UCL students and the UK context and therefore applies to the CAP-2030 secretariat as UCL employees. The policy states important child protection principles:

- The definition of a child is anyone under 18 years old
- Safeguarding is the responsibility of all UCL staff
- There is a duty to report a concern regarding the safety of a child and a specific procedure to be followed
- Records should be kept of any safeguarding reports and the actions taken
- If a child is in danger, information should be shared with the relevant authorities (this may take place without consent if in the best interests of the child).

2. Context and underlining principles

2.1 Positive safeguarding culture

We will strive to build a culture where safeguarding is practiced in the context of a positive environment and concern with the wellbeing of children. Children and their safety are viewed holistically and in all activities, not exclusively when there is a concern of abuse.

As a global network, we recognise that there are diverse laws and practices in different countries. We respect local contexts and national policies. If concerns arise, we will consult partners in local contexts to ensure the appropriate support is provided to children.

2.2 Best interests of the child

Article 3 of the UNCRC states, “In all actions concerning children, whether undertaken by public or private social welfare institutions, courts of law, administrative authorities or legislative bodies, the best interests of the child shall be a primary consideration.” We will uphold this principle in all areas of work.

2.3 Relationships with a child’s parent or guardian

The UNCRC recognises the role of children’s parents or guardians as children’s primary care givers and protectors. CAP-2030 respects the essential role of parents and guardians in children’s lives and will seek to inform and involve them in activities related to their children whenever possible. This will normally involve seeking written consent to take part in activities from parents or guardians as well as children. However, the form and way consent is obtained will be adapted according to the needs of the child and their primary care giver, and the context in which an activity takes place.

3. Creating a safe organisation

3.1 Work with partners

We work with trusted partners who have experience working with children and young people. Continued close relationships and open communication with partners is crucial as we seek to strengthen and uphold safeguarding practices. We are committed to understanding culturally appropriate ways of safeguarding children.

It is important that partners have functioning safeguarding practices in place to protect the children they work with. Partners should have an overall understanding of risks to children from their organisations and must have measures in place to address those risks. These measures may include:

- A functioning child protection/ safeguarding policy which includes:
    - A system for reporting and processing any child protection concerns.
    - A code of conduct for staff’s interaction with children.
    - Procedures regarding safe recruitment.

- An expectation, and resources, for staff to receive the appropriate training to adhere to the policy.

We would expect to have access to partners' relevant safeguarding policies and documents. The organisation leading safeguarding will be agreed before work commences.

We will endeavour to work with partners to ensure that these measures are in place and functioning. A list of useful resources for partners is provided in the appendix.

3.2 Respectful communication

We are committed to ensuring that all communication and online spaces respect children's dignity and do not exploit their vulnerabilities or that of their families and communities.

Organisations will:

- Ensure all communication respects the dignity of children.
- Use images truthfully.
- Use images that celebrate children's diversity and strengths.

Organisations will not:

- Use images that children and their guardians have not consented to have used.
- Include pictures or case studies that are offensive, shocking or portray children as powerless victims.
- Compound discrimination through stereotyped images or descriptions of children's cultures, identities, or abilities.

3.3 Events, activities and consultations including children

We seek to amplify children's voices in various ways, including by involving them in events and consultations. Whenever children are included in these activities we will:

- Obtain informed consent from the child and their primary care giver.
- Ensure their views are listened to and respected.
- Identify potential risks in advance and work towards mitigating these.
- Consider the wellbeing of the child as paramount, which will involve:
    - Ensuring the necessary support is available for them.
    - Including a known and responsible adult in the organisation of, and communication regarding, the activity.
    - Providing information in advance.
    - Ascertaining the child's needs and ensuring they are met.
    - Ensuring the child and responsible adult know how and where to raise concerns.

When involving child participation in online activities we will follow the recommendations outlined in the NSPCC guidelines on Social media and online safety

4. Code of conduct

The CAP-2030 team will adhere to the following behaviours.

- We will always treat a child with respect and dignity;
- We will treat all children equally;
- We will work to empower children, respect their views and provide spaces for their voices to be heard;
- We will ensure child-friendly language and communication are used;
- We will endeavour to meet children's individual needs and make activities accessible for them;
- We will respect and consider a child's cultural context;
- We will respond to concerns regarding children's wellbeing appropriately;
- We will report any abuse of a child, disclosed or witnessed, to the appropriate authorities;
- We will not act in a way that communicates favouring one child above another;
- We will not use images of children without their and their care giver's consent;
- We will not degrade, belittle or knowingly embarrass a child;
- We will not use language or show images to a child that are inappropriate for their age or maturity level;
- We will not seek to be alone with a child or form an inappropriate personal relationship with a child.

5. Recognising and responding to concerns

5.1 Recognising where there is a concern and understanding signs of abuse

When working closely with a child, concerns may arise that they are experiencing harm. We are committed to considering all concerns carefully. When working cross-culturally we should seek advice, without disclosing a child's identity, from experts within the child's cultural context to ensure they have not misunderstood signs of abuse.

Here are some signs that abuse might have occurred[1]:

- Unexplained injuries or regular injuries
- Unexplained changes in behaviour
- A child becomes withdrawn or seems anxious
- Uncharacteristic aggression
- Knowledge of adult issues inappropriate for their age
- Running away or going missing

[1] Per the website of the National Society for the Prevention of Cruelty to Children, [Spotting the signs of child abuse | NSPCC]

5.2 Responding to a disclosure of abuse

How adults respond to children's disclosure of abuse has an important impact on children's emotional wellbeing. Children who have experienced abuse, can suffer further trauma if they feel that their disclosures are not believed or taken seriously.

If a child discloses abuse you should:

- Listen empathetically. Allow the child to speak, show that you care and are concerned.
- Reflect what the child is telling you and show you understand. Do not add words to their disclosure or make assumptions.
- Do not interrogate or ask questions about details. This is not your role and is likely to make the child feel that they are not believed.
- Do not promise confidentiality but explain that you will need to let people know who can help them and not anyone else.
- Explain what you will do next.

5.3 Who to report an incident or concern to

Incidents and concerns should be reported to senior management or a safeguarding lead unless they are directly involved. In this case, the report should be made to senior member of management at the UCL Institute for Global Health or appropriate organisation. If a child is in immediate danger, the relevant authorities should be contacted.

5.4 How to record an incident or concern

All incidents or concerns should be recorded. The information recorded by the person who witnessed a concern or heard a disclosure should include:

- Child's name
- Dates
- Description of incident/s or concern
- Accurate details of what was said
- Name and details of people involved (specifically perpetrator)
- Child's current location and any concerns for their immediate safety
- Any other relevant information.

When CAP-2030 is directly involved, the management will maintain a record of all actions taken in response to the concerns. Records will be stored securely. Records and information about the case will only be shared on a need-to-know basis, with the safety of the child as the determinate of what and how the information should be shared.

5.5 How an incident or concern will be dealt with

The wellbeing and safety of the child will be paramount in all responses to concerns regarding a child. When CAP-2030 is directly involved, the Executive Director will be responsible for contacting and coordinating a response with the appropriate partners and authorities.

Appendix: List of Safeguarding Resources

Organisation	Source / Link
University College London	Safeguarding Policy: https://www.ucl.ac.uk/human-resources/safeguarding-children-and-adults-risk-policy-and-procedure-staff-and-students#Safeguarding%20Students%20Under%2018-%20Preventative%20Action<br><br>Social Media Guidance: https://www.ucl.ac.uk/cam/social-media-guide/guidelines-staff
Child Rights Connect Safeguarding Policy	https://www.childrightsconnect.org/wp-content/uploads/2021/08/crcnct-child-safeguarding-policy-procedure.pdf
Keeping Children Safe Coalition	www.keepingchildrensafe.org.uk<br>International Child Safeguarding Standards (KCS 2018a)<br>Child Safeguarding Standards and how to implement them (KCS 2014a)<br>Self-audit tool: Is your organisation doing all it can to keep children safe? (KCS 2018b)<br>Developing a Child Safeguarding Policy and Procedures: A Facilitator's guide (KCS 2014b)<br>Code of Good Practice in the management and support of aid personnel (KCS 2003)<br>International Safeguards for Children in Sport (KCS 2018c)<br>Keeping Children Safe Online: a guide for organisations (2014c)<br>Child safeguarding guidance for journalists and filmmakers (2014d)
Ethical Research Involving Children (ERIC)	Guidance: https://childethics.com/ethical-guidance/
Terre des Hommes	Child Safeguarding Policy: https://www.tdh.ch/sites/default/files/955ede1a-c959-4e91-914e-5aa7fb944b28_r_pse_d_pol_en_original_0.pdf
PLAN	Global Policy on Safeguarding Children and Young People | Plan International (plan-international.org)
UNICEF UK	Our statement on safeguarding - Unicef UK<br>Safeguarding-Policy-UnicefUK.pdf
Council of Europe	16808c8b91 (coe.int)

Child Rights International (CRIN) Child safeguarding policy and procedures	https://static1.squarespace.com/static/5afadb22e17ba3eddf90c02f/t/607465f94a004d410d2c4bc1/1618241018299/CRIN_+Child+Safeguarding+and+Protection+Policy+web.pdf
Gaia Education	Gaia Education Policies – GaiaEducation.org<br><br>Organisational Structure note: They have core staff and about 142 partner organisations across the globe.
NSPCC	https://learning.nspcc.org.uk/safeguarding-child-protection<br><br>Social Media and online safety: https://learning.nspcc.org.uk/safeguarding-child-protection/social-media-and-online-safety
