# Supplementary material for: Involving children in global health policy and programming: practical guidance to get started
Source: Health Promot Int. 2025 Apr 23;40(2):daaf041. doi: 10.1093/heapro/daaf041 (PMC12015606; doi:10.1093/heapro/daaf041)
Supplement: daaf041_suppl_Supplementary_Material [file daaf041_suppl_supplementary_material.zip › CAP-2030_YAB_Privacy_Notice_vf.pdf]

CAP-2030 Youth Advisory Board Privacy Notice

1. Introduction

1.1 Introduction to CAP-2030 and purpose of the Privacy Notice

CAP-2030's mission is to centre children's wellbeing and health at the heart of all policies. As we strive to create a better world for children globally, it is essential to ensure the safety and wellbeing of the children we have relationships with, either through direct interaction or via partnerships. CAP-2030 is committed to being a safe organisation for all children.

CAP-2030 respects your privacy and is committed to protecting your personal data

Please read this Privacy Notice carefully – it describes why and how we collect and use personal data and provides information about your rights. It applies to personal data provided to us, both by individuals themselves or by third parties and supplements the following wider UCL privacy notice(s):

- General privacy notice when you visit UCL's website
- Student privacy notice
- Staff privacy notice
- Research participants for health and care purposes privacy notice

Furthermore, it sets out our standards and expected practices for CAP-2030's partners and networks.

2. Who we are

2.1 About us

We are Children in All Policies 2030 (CAP-2030), a group based at the Institute for Global Health at University College London (UCL).

UCL, a company incorporated by Royal Charter (number RC 000631), is the entity that determines how and why your personal data is processed. This means that UCL is the 'controller' of your personal data for the purposes of data protection law.

We bring together people from all over the world to improve Children's Health and Well-being. Some of the key areas of concern for us are:

- Climate change
- Harmful commercial marketing (organisations that sell products to children that are harmful to their health)
- Using data (information) to improve policies that impact children.

We work with researchers and organisations who are experts in their subjects and we recognise the importance of involving young people and learning from their lived experiences. Involving young people will give us a better understanding of the issues you face; your priorities and perspective. That is why we have formed the Youth Advisory Board (YAB).

To find out more about who we are and what we do please check out our website and social media accounts on Instagram and Twitter.

2.2 How to get in touch with the CAP-2030 YAB team.

CAP-2030 YAB Staff

Beth Jennings
CAP-2030 Youth Advisory Board Lead
Email: bethany.jennings@ucl.ac.uk
Tel:

Sarah Sterlini
CAP-2030 Youth Advisory Board Co-ordinator
Email: s.sterlini@ucl.ac.uk
Tel: +44 20 8138 7917

Sarah Dalglish
Executive Director, CAP-2030
Email: s.dalglish@ucl.ac.uk

Write to Institute for Global health, 3rd Floor, Institute of Child Health, 30 Guilford Street, London, WC1N 1EH

3. Personal data

3.1 Personal data we collect about you

Personal data, or personal information, means any information about an individual from which that person can be identified. It does not include data where the identity has been removed (anonymous data).

We may collect, use, store and transfer different kinds of personal data about you. This may include:

- name, contact details (email, phone and address) for CAP-2030 YAB members and if under 18 years old, parents and guardians.
- social media handles for CAP-2030 YAB members
- photos, stories, comments and videos made by CAP-2030 YAB members and/or UCL staff about individuals participation in CAP-2030 YAB activities and the wider work of the CAP-2030 initiative.
- information about CAP-2030 YAB members backgrounds, relevant to the work of the CAP-2030 YAB (for example, links to other organisations or previous advocacy work around similar issues).
- feedback from CAP-2030 YAB members about CAP-2030 YAB activities, the wider work of the CAP-2030 initiative (i.e. our working groups) and our collaborating partners and stakeholders.
- feedback from CAP-2030 YAB members and/or their parents/guardians regarding CAP-2030 YAB activities, systems, processes and procedures.

3.2 How we use your personal data

We will only use your personal data when the law allows us to. Most commonly, we will use your personal data in the following circumstances:

- to register you as CAP-2030 YAB members, consider you for related groups/specific activities and to manage our relationship with you.
- to help you with any queries or concerns which are raised during or in relation to CAP-2030 YAB activities. Depending on the circumstances, this may include special category personal data. Here, the processing of your information is carried out on the basis of your explicit consent.
- to check that the content we send you is age appropriate, for example, is the language used child-friendly?, is the information provided likely to upset a young audience?

- if you are under the age of 18, to obtain parental/guardian consent, keep parents/guardians informed of CAP-2030 YAB activities and register contact details in case of emergency.
- to send you updates about CAP-2030 YAB activities and the wider work of the CAP-2030 initiative (for example, if you've signed up to our newsletter)
- amplify our social media messaging for example, if you interact with us in person, on our website or via our social media we may share your comments with our audiences, and collaborating partners/stakeholders
- develop content (Website profiles, blogs, news articles, social media posts) promoting CAP-2030 YAB activities and the work of the wider initiative
- work with our collaborating partners and key stakeholders to promote the work of the CAP-2030 YAB and wider initiative, at our events, online and in the media
- keep track of the progress of CAP-2030 YAB activities, to monitor and report impact
- to enable you to partake in our prize draws or competitions
- to maintain and improve our systems, processes and procedures use your feedback to help us determine our priorities.

Asking for consent

If we need to, we will ask you and/or your parent/guardian for consent (This means ask your permission) to use your personal information. We will send you and your parents a consent form to sign, with a clear description of the personal information we would like to collect, why and how we will use and store it. This information is then stored on secure UCL systems to ensure we keep your personal information safe.

Where the processing is based on your consent, **you have the right to withdraw your consent at any time** by contacting the CAP-2030 YAB team (details on p2) and the UCL Data Protection Officer (details on p7) Please note that this will not affect the lawfulness of processing based on consent before its withdrawal.

We may also use anonymised data, meaning data from which you cannot be identified, for the purposes of:

- Service evaluation;
- Education and research; or
- Fundraising and promotional purposes.

Anonymised data may also be used in published reports or journals and at conferences.

3.3 Who we share your personal data with

Your personal data will be collected and processed primarily by our staff and UCL (Access to your personal information is limited to staff who have a legitimate need to see it for the purpose of carrying out their job at UCL and CAP-2030 governance and activities.). We may have to share your personal data with the parties set out below for the purposes outlined in section 4:

[Please list all third party organisations: UNICEF, WHO, University of the Western Cape (UWC), Center for Learning and Childhood Development (CLCD), Non-Communicable Disease Alliance (NCDA), Kids4SDGs, Learning Planet
We require all third parties to respect the security of your personal data and to treat it in accordance with the law. We do not allow our third party service providers to use your personal data for their own purposes – we only permit them to process your personal data for specified purposes and in accordance with our instructions.

3.4 Lawful Basis for Processing

Data Protection Legislation requires that we meet certain conditions before we are allowed to use your data in the manner described in this notice, including having a "lawful basis" for the processing. The basis for processing will be as follows:

- Public task. The processing of your personal data may be necessary for the performance of a task carried out in the public interest or in the exercise of official authority vested in us **[Note: read UCL's Statement on Public Tasks to ensure that your processing falls within scope of this condition]**

- Consent. In some cases we may request your consent for processing your personal data.

4. Keeping your data safe

4.1 International transfers

We do not transfer your personal data outside the United Kingdom or the European Economic Area (**EEA**).

4.2 Information security

We have put in place appropriate security measures to prevent your personal data from being accidentally lost, used or accessed in an unauthorised way, altered or disclosed. We have established procedures to deal with any suspected personal data breach and will notify you and any applicable regulator of a breach where we are legally required to do so.

5. Data Retention

5.1 How long do we keep your data

We will only retain your personal data for as long as necessary to fulfil the purposes we collected it for, including for the purposes of satisfying any legal, accounting, or reporting requirements.

We will keep your personal data according to the Records Retention Schedule.

6. Data Protection Legislation

6.1 Your Rights

Under certain circumstances, you may have the following rights under data protection legislation in relation to your personal data:

- Right to request access to your personal data;
- Right to request correction of your personal data;
- Right to request erasure of your personal data;
- Right to object to processing of your personal data;
- Right to request restriction of the processing your personal data;
- Right to request the transfer of your personal data; and
- Right to withdraw consent.

If you wish to exercise any of these rights, please contact a member of CAP-2030 YAB staff (Contact details Page 2 & 3, Section 2.2). and the **UCL Data Protection Officer**

Please note that UCL has appointed a Data Protection Officer. If you have any questions about this Privacy Notice, including any requests to exercise your legal rights, please contact our Data Protection Officer using the details set out below:

Data Protection & Freedom of Information Officer
data-protection@ucl.ac.uk

You can contact UCL by telephoning +44 (0)20 7679 2000 or by writing to: University College London, Gower Street, London WC1E 6BT.

7. Complaints

7.1 Who do I speak to if I have a complaint?

If you wish to complain about our use of personal data, please send an email with the details of your complaint to a member of CAP-2030 YAB staff and the UCL Data Protection Officer so that we can look into the issue and respond to you.

You also have the right to lodge a complaint with the Information Commissioner's Office (**ICO**) (the UK data protection regulator). For further information on your rights and how to complain to the ICO, please refer to the ICO website.

8. Other important information

8.1 Links to other websites and social media activity

This privacy notice only covers how CAP-2030 uses information about you, it does not cover other websites or social media sites that we might use or share with you or that you might use to interact with us. They each have their own rules so make sure you read the privacy notices on sites/platforms you visit to make sure you understand how they use information about you.

It is important that we stay safe when engaging in online activities and to be aware of the potential risks. We recommend the safety guidance below for further information:

For children

- NSPCC Staying Safe Online
- NSPCC Guidance on how to feel good on social media

For Parents

- NSPCC Online Safety Guides for parents
- NSPCC Guidance on keeping children safe on social media

If at any point, you feel unsafe engaging in online activities associate with the CAP-2030 YAB, please contact a member of our staff and/or tell a parent/guardian or a trusted adult. **Your safety, health and well-being is our priority.**

8.2 Updates

If we update this 'privacy notice' in any way that significantly changes the way, we use your personal information, we will let you know as soon as possible.
